# Correction: Understanding knowledge, attitude and perception of Rift Valley fever in Baringo South, Kenya: A cross-sectional study

**DOI:** 10.1371/journal.pgph.0003400

**Published:** 2024-06-13

**Authors:** The PLOS Global Public Health Staff

There are errors in the author affiliations. The correct affiliations are as follows:

Tatenda Chiuya^1^, Eric M. Fevre^2,3^, Sandra Junglen^4^, Christian Borgemeister^1^

**1** Centre for Development Research (ZEF), University of Bonn, Bonn, Germany, **2** International Livestock Research Institute (ILRI), Nairobi, Kenya, **3** Institute of Infection, Veterinary and Ecological Sciences, University of Liverpool, Leahurst Campus, Neston, United Kingdom, **4** Institute of Virology, Charité—Universitätsmedizin Berlin, Corporate Member of Free University Berlin, Humboldt-University Berlin and Berlin Institute of Health, Berlin, Germany.

The publisher apologizes for the error.
