# Supplementary material for: Understanding knowledge, attitude and perception of Rift Valley fever in Baringo South, Kenya: A cross-sectional study
Source: PLOS Glob Public Health. 2023 Sep 12;3(9):e0002195. doi: 10.1371/journal.pgph.0002195 (PMC10497146; doi:10.1371/journal.pgph.0002195)
Supplement: S1 Questionnaire — (PDF) [file pgph.0002195.s001.pdf]

S1 PDF: Arbovirus questionnaire

Get GPS coordinates

latitude (x.y °)

longitude (x.y °)

altitude (m)

accuracy (m)

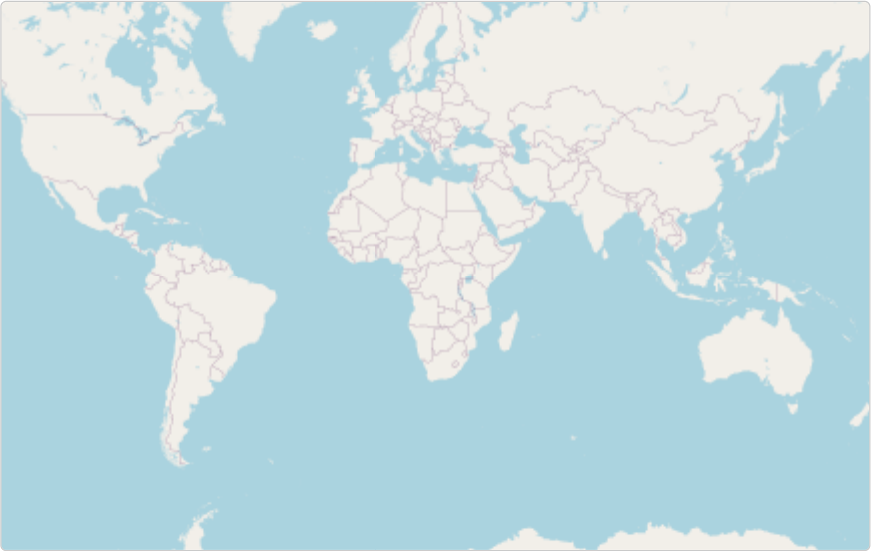

Dear Sir/Madam, I work for the International Livestock Research Institute and University of Bonn. We are conducting a survey in Baringo County to understand the knowledge, perception and coping strategies of agro-pastoralists on arboviral diseases. All the data disclosed during this interview will remain confidential to the research team. Your participation is voluntary and if you consent and then later decide that you would like to withdraw, you are free to do this at any point.

Initials of enumerator

Do you consent to participate in this survey?

- Yes
- No

A. SITE AND HOUSEHOLD CHARACTERISTICS

Name of household head

Enter full name(firstname, surname)

Name of respondent if its not the household head being interviewed

If its household head responding enter NA

Relationship of respondent to the household head

If its household head responding enter NA

- ◯ Wife
- ◯ Daughter
- ◯ Son
- ◯ Brother
- ◯ Sister
- ◯ Cousin
- ◯ Son in-law
- ◯ Daughter in-law
- ◯ Grandchild
- ◯ Nephew
- ◯ Niece
- ◯ Helper
- ◯ NA

Gender of the household head

- ◯ Male
- ◯ Female

Age of household head

If exact age is unknown enter an estimate

____

Tribe of the household head

____

Sub-tribe of the household head

____

Location

____

Sub-location

____

Village

____

Was the household head born and raised in this locality?

- ◯ Yes
- ◯ No

From where did they migrate to this village?

- ◯ From another village within the sub-location
- ◯ From another sub-location
- ◯ From another county
- ◯ From another country

Name of the place they migrated from?

Which year did they migrate to the locality?

If not sure estimated year of migration is acceptable

What was the main reason for migration?

Religion of the household head

- ◯ Catholic
- ◯ Protestant (all non-catholic)
- ◯ Muslim
- ◯ ATR
- ◯ Non-religious

Marital status of household head

- ◯ Married
- ◯ Single
- ◯ Divorced
- ◯ Widowed

Number of household members

Household head level of formal education

- ◯ None
- ◯ Primary
- ◯ Secondary
- ◯ Post-secondary tertiary
- ◯ University

What is your household's main economic activity/source of income?

- ◯ Farming (crop/livestock)
- ◯ Salaried employment
- ◯ Self-employed (off-farm)
- ◯ Casual laborer on-farm
- ◯ Casual laborer off-farm
- ◯ Other

Specify(other)

____

What are the other supplementary sources of income in the household?

If there is none enter NA

____

Type of livestock owned by household

- ☐ Cattle
- ☐ Goats
- ☐ Sheep
- ☐ Camels
- ☐ Donkeys
- ☐ Pigs
- ☐ Poultry
- ☐ Rabbits
- ☐ Horses

How many cattle do you have?

____

How many goats do you have?

How many sheep do you have?

How many camels do you have?

How many donkeys do you have?

How many pigs do you have?

Number of poultry?

How many rabbits do you have?

How many horses do you have?

B. HOUSE CONDITION

Do you have a permanent dwelling?

- Yes
- No

Material making up the house

Which roofing material is on the main house?

This can also be observed if interview is being done at homestead

- ◯ Tiles
- ◯ Asbestos
- ◯ Tin
- ◯ Iron sheets
- ◯ Concrete
- ◯ Thatch
- ◯ Other

Specify(other)

What type of floor is in the main house?

- ◯ Tiles
- ◯ Cement
- ◯ Wood
- ◯ Sand
- ◯ Earth/Soil

What material makes up the walls of the main house?

This can also be observed if interview is being done at homestead

- ◯ Mud&poles
- ◯ Brick&cement
- ◯ Iron sheets
- ◯ Unburnt bricks&mud
- ◯ Grass&poles

Electronics

Do you have a TV?

- ◯ Yes
- ◯ No

Do you have a radio?

- ◯ Yes
- ◯ No

Do you have a computer?

- ◯ Yes
- ◯ No

Do you have a smartphone?

- ◯ Yes
- ◯ No

What is the main source of energy for cooking in the household?

The main source

- ◯ Electricity
- ◯ Solar
- ◯ Generator
- ◯ Gas
- ◯ Kerosene
- ◯ Firewood
- ◯ Charcoal

What is the main source of energy for lighting in the household?

The main source

- ◯ Candle
- ◯ Electricity
- ◯ Solar
- ◯ Generator
- ◯ Torch
- ◯ Gas
- ◯ Kerosene

What is the main source of water used for consumption in the house?

The main source

- Piped water
- Protected well
- Unprotected well
- Spring
- Dam
- Lake
- River
- Canal
- Other

Specify(other)

What type of toilet is used by the household?

- Flush
- Blair/Pit with slab
- Pit without slab
- No facility

C. ECOLOGICAL SETTING AND PROXIMITY TO SOCIAL AMENITIES

What is the distance from the homestead to the nearest health facility?

Distance estimated in kilometres

What is the distance from homestead to the nearest primary school?

Distance estimated in kilometres

What is the distance from homestead to the nearest secondary school?

Distance estimated in kilometres

What is the distance from homestead to a conservation area?

Distance estimated in kilometres

Which conservation area is close to your locality?

- National park
- Community conservancy
- Forest reserve
- Game reserve
- Private conservancy

Name of the conservancy

Is this conservation area fenced or is there a physical separating barrier?

- Yes
- No
- I don't know

What is the state of the fence/barrier?

Very bad | Rather bad | Passable | Rather good | Very good

Is there any form of community-based natural resource management in this conservation area?

- Yes
- No
- I don't know

Are you involved in this community-based natural resource management in this conservation area?

- Yes
- No

How are you involved in this community-based natural resource management?

Role/position held by the respondent

Overall, how do you evaluate the impact of conservation on your household's well-being?

Very Negative | Negative | Neutral | Positive | Very Positive

Overall, how do you evaluate the impact of conservation on your community's well-being?

Very Negative | Negative | Neutral | Positive | Very Positive

Do your livestock mix with wild animals?

- Yes
- No
- I don't know

Where do they mix?

- Watering points
- Grazing lands
- Households
- Other

Specify(other)

Which species of wildlife have they mixed with?

Allow respondent to list all and enter the species separated by commas

Do wildlife come close to your homesteads?

- Yes
- No
- I don't know

Which species?

Allow respondent to list all and enter the species separated by commas

In the past, have you experienced any wildlife conflicts?

- Yes
- No

What kind of damages were incurred?

- Crop damage
- Loss of livestock
- Destruction of property
- Injury to self/family members

Which wildlife species were responsible?

Allow respondent to list all and enter the species separated by commas

Do you think there has been a change in wildlife populations in the area?

- Increase
- No change
- Decrease
- I don't know

Which species of wildlife has changed?

Present question if previous answer was increase/decrease otherwise enter NA

Do you think there has been a change in the vegetation cover in the area?

- Increase
- No change
- Decrease
- I don't know

Which type of vegetation has increased?

D. KNOWLEDGE ABOUT RIFT VALLEY FEVER IN HUMANS

Have you heard of Rift Valley fever disease before in humans?

Be clear you are referring to RVF in humans not in animals. If answer is NO proceed to section F. (Dengue fever)

- Yes
- No

Knowledge of Rift Valley fever

How/where did you learn about Rift Valley fever disease in humans?

Allow respondent to list while you tick. If not on list use "other" option

- [ ] Health professional
- [ ] Veterinary professional
- [ ] Social media
- [ ] Poster/pamphlets
- [ ] Book
- [ ] Radio/Television
- [ ] Friends/relatives
- [ ] Church/Mosque
- [ ] NGOs/researchers
- [ ] Community gathering
- [ ] Patients who suffered from the disease
- [ ] I don't know
- [ ] Other

Specify(other)

Does Rift Valley fever also affect animals?

- ◯ Yes
- ◯ No
- ◯ I don't know

Have you or any of your household members suffered from Rift Valley fever?

- ◯ Yes
- ◯ No

Has any member of your household died from Rift Valley fever?

- ◯ Yes
- ◯ No

What kind of clinical signs/symptoms do you associate with Rift Valley fever in humans?

Allow respondent to list while you tick. If not on list use "other" option

- [ ] Fever
- [ ] Headache
- [ ] Muscle pain
- [ ] Joint pain
- [ ] Back ache
- [ ] Rash
- [ ] Weakness
- [ ] Vomiting
- [ ] Diarrhoea
- [ ] Abdominal/gastric discomfort
- [ ] Tremors
- [ ] Haematemesis (Vomiting blood)
- [ ] Epistaxis (Nose bleeding)
- [ ] Gum bleeding
- [ ] Haematochezia (Blood in faeces)
- [ ] Abortion
- [ ] Eye pain and congestion (Redness)
- [ ] Cough
- [ ] Dizziness
- [ ] Coma
- [ ] I don't know
- [ ] Other

Specify(other)

Which season do you associate with the occurrence of Rift Valley fever?

How does a person get infected with Rift Valley fever?

E. ATTITUDES TO RIFT VALLEY FEVER IN HUMANS

Is Rift Valley fever a serious health problem in people in your area?

Select one

- Strongly agree
- Agree
- Neither agree nor disagree
- Disagree
- Strongly disagree

What will you do in the event of illness from Rift Valley fever?

- Go to the hospital
- Buy medicine from pharmacy
- Do nothing
- Go to traditional healer
- Use herbs to treat myself
- Consult prophet at church
- Other

Specify(other)

Give a reason for your selected course of action?

This question is based on the "action" they take in previous question

How effective do you think this course of action will be in resolving the illness from Rift Valley fever?

This question is based on the "action" they take in previous question

- Not effective at all
- Little effective
- Somehow effective
- Effective
- Very effective

How do you think the occurrence of Rift Valley fever in people has changed over the past years?

- Increased
- Decreased
- No change
- Don't know

What do you think has changed the occurrence of Rift Valley fever in people?

Present question if answer for previous question is increased/decreased otherwise enter NA

F. KNOWLEDGE ABOUT DENGUE FEVER

Have you heard of Dengue fever before?

If answer is NO proceed to section H. (RVF in animals)

- Yes
- No

Knowledge of Dengue fever

How/where did you learn about Dengue fever?

Allow respondent to list while you tick. If not on the list use "other" option

- ☐ Health professional
- ☐ Veterinary professional
- ☐ Social media
- ☐ Poster/pamphlets
- ☐ Book
- ☐ Radio/Television
- ☐ Friends/relatives
- ☐ Church/Mosque
- ☐ NGOs/researchers
- ☐ Community gathering
- ☐ Patients who suffered from the disease
- ☐ I don't know
- ☐ Other

Specify(other)

Do you think Dengue fever affects domestic animals?

- ○ Yes
- ○ No
- ○ I don't know

Have you or your household member suffered from Dengue fever?

- ○ Yes
- ○ No

Has any member of your household died from Dengue fever?

- ○ Yes
- ○ No

What kind of clinical signs/symptoms do you associate with Dengue fever in humans?

Allow respondent to list while you tick. If not on the list use "other" option

- [ ] Fever
- [ ] Headache
- [ ] Muscle pain
- [ ] Joint pain
- [ ] Back ache
- [ ] Rash
- [ ] Weakness
- [ ] Vomiting
- [ ] Diarrhoea
- [ ] Abdominal/gastric discomfort
- [ ] Tremors
- [ ] Haematemesis (Vomiting blood)
- [ ] Epistaxis (Nose bleeding)
- [ ] Gum bleeding
- [ ] Haematochezia (Blood in faeces)
- [ ] Abortion
- [ ] Eye pain and congestion (Redness)
- [ ] Cough
- [ ] Dizziness
- [ ] Coma
- [ ] I don't know
- [ ] Other

Specify(other)

____

Which season do you associate with the occurrence of Dengue fever?

____

How does a person get infected with Dengue fever?

Ask respondent to answer before reading out options. If not on the list use "other" option

- ◯ Mosquito bites
- ◯ Contact with infected domestic animals
- ◯ Contact with wildlife
- ◯ Handling arbotus
- ◯ Eating raw infected meat
- ◯ Drinking raw milk
- ◯ Drinking raw blood
- ◯ Contact with blood and tissues of infected animal during slaughter/skinning
- ◯ I don't know
- ◯ Other

Specify(other)

G. ATTITUDES TO DENGUE FEVER

Is Dengue fever a serious health problem in your area?

Select one

◯	◯	◯	◯	◯
Strongly agree	Agree	Neither agree nor disagree	Disagree	Strongly disagree

What would you do in the event of illness from Dengue fever?

- ◯ Go to the hospital
- ◯ Buy medicine from pharmacy
- ◯ Do nothing
- ◯ Go to traditional healer
- ◯ Use herbs to treat myself
- ◯ Consult prophet at church
- ◯ Other

Specify(other)

Give a reason for your selected course of action?

This question is based on the "action" they take in previous question

How effective do you think this course of action is in resolving the illness from Dengue fever?

This question is based on the "action" they take in previous question

- Not effective at all
- Little effective
- Somehow effective
- Effective
- Very effective

How do you think the occurrence of Dengue fever in this area has changed over the past years?

- Increased
- Decreased
- No change
- Don't know

What do you think has changed this occurrence of Dengue fever?

Present question if answer for previous question is increased/decreased otherwise enter NA

H. KNOWLEDGE ABOUT RIFT VALLEY FEVER IN ANIMALS

Have you heard of Rift Valley fever disease in animals before?

Be clear you are referring to RVF in animals.

- Yes
- No

Knowledge of Rift Valley fever in animals

How did you hear about Rift Valley fever in animals?

Allow respondent to list while you tick. If not on list use "other" option.

- [ ] Health professional
- [ ] Veterinary professional
- [ ] Social media
- [ ] Poster/pamphlets
- [ ] Book
- [ ] Radio/Television
- [ ] Friends/relatives
- [ ] Church/Mosque
- [ ] NGOs/researchers
- [ ] Community gathering
- [ ] Other

Specify(other)

Has any of your animals suffered from Rift Valley fever?

- ◯ Yes
- ◯ No

Has any of your animals died from Rift Valley fever?

- ◯ Yes
- ◯ No

What are the clinical signs and symptoms of Rift Valley fever in animals?

Allow respondent to list while you tick. If not on list use "other" option.

- ☐ Fever
- ☐ Lack of appetite
- ☐ Abortion
- ☐ Nasal discharge
- ☐ Ocular(eye) discharge
- ☐ Salivation
- ☐ Diarrhoea with blood
- ☐ Loss of weight
- ☐ Muscle stiffness
- ☐ Lameness
- ☐ Recumbency (Lying down)
- ☐ Decrease in milk production
- ☐ Facial oedema (Swelling)
- ☐ Oral lesions and ulcers
- ☐ Mucosal congestion (Reddening)
- ☐ I don't know
- ☐ Other

Specify(other)

Which domestic animals are commonly affected by Rift Valley fever?

- [ ] Cattle
- [ ] Sheep
- [ ] Goats
- [ ] Pigs
- [ ] Camels
- [ ] Donkeys
- [ ] Rabbits
- [ ] Chicken and other poultry
- [ ] Calves
- [ ] Lambs
- [ ] I don't know

Where do you think your animals pick Rift Valley fever infection from?

Allow respondent to list while you tick before reading out options. If not on the list use "other" option

- [ ] Lake/watering places
- [ ] Conservation areas
- [ ] Grazing lands
- [ ] Agriculture plots
- [ ] Bushes
- [ ] Other herds
- [ ] Homesteads
- [ ] Other
- [ ] I don't know

Specify(other)

How is Rift Valley fever transmitted to animals?

Allow respondent to list while you tick. If not on list use "other" option.

- [ ] Mosquito bites
- [ ] Contact with abortus in pasture
- [ ] Contact with infected animal
- [ ] Drinking water contaminated by infected animals
- [ ] Contact with wildlife
- [ ] Direct contact with discharge from infected animal
- [ ] Other
- [ ] I don't know

Specify(other)

I. ATTITUDES TO RIFT VALLEY FEVER IN ANIMALS

Do you think Rift Valley fever is a threat to the health of your animals?

- ○ Strongly agree
- ○ Agree
- ○ Neither agree/disagree
- ○ Disagree
- ○ Strongly disagree

What action would do you take if your animal is sick from Rift Valley fever?

- ○ Do nothing
- ○ Slaughter for meat
- ○ Buy medicines from Agrovet and treat
- ○ Call veterinary practitioner
- ○ Ask for advice from medical practitioner
- ○ Treat with local herbs
- ○ Other

Specify(other)

How do you assess the effectiveness of the action you take?

This question is based on the "action" they take in previous question

- Not effective at all
- Little effective
- Somehow effective
- Effective
- Very effective

What action would do you take if your animal dies from Rift Valley fever?

- Bury the carcass
- Use the meat for consumption
- Sell the meat
- Give it to dogs
- Nothing/leave it to decompose
- Other

Specify(other)

How has the occurrence of Rift Valley fever in animals in this area changed in the past years?

- Increased
- Decreased
- No change
- I don't know

What do you think has changed this occurrence of Rift Valley fever?

Present this question if previous answer is increased/decreased otherwise enter NA

Risk factors for Rift Valley fever in humans and animals

J. RISK FACTORS FOR RIFT VALLEY FEVER IN HUMANS AND ANIMALS

The presence/ increase in population of wild animals is a risk factor for RVF

- Strongly disagree
- Disagree
- Neither disagree/agree
- Agree
- Strongly agree

Flooding is a risk factor for Rift Valley fever

- Strongly disagree
- Disagree
- Neither disagree/agree
- Agree
- Strongly agree

Bushy vegetation is a risk factor for Rift Valley fever

- Strongly disagree
- Disagree
- Neither disagree/agree
- Agree
- Strongly agree

Drought is a risk factor for Rift Valley fever

- Strongly disagree
- Disagree
- Neither disagree/agree
- Agree
- Strongly agree

Irrigation on agricultural plots is a risk factor for Rift Valley fever

- Strongly disagree
- Disagree
- Neither disagree/agree
- Agree
- Strongly agree

Increasing cattle population is a risk factor for Rift Valley fever

- Strongly disagree
- Disagree
- Neither disagree/agree
- Agree
- Strongly agree

Increasing human population is a risk factor for Rift Valley fever

- Strongly disagree
- Disagree
- Neither disagree/agree
- Agree
- Strongly agree

Contact with animals during herding, milking, birthing, slaughtering and butcher of livestock is a risk factor for Rift Valley fever

- Strongly disagree
- Disagree
- Neither disagree/agree
- Agree
- Strongly agree

Human consumption of undercooked/raw livestock products is a risk factor for Rift Valley fever

- Strongly disagree
- Disagree
- Neither disagree/agree
- Agree
- Strongly agree

K. KNOWLEDGE ON THE VECTORS OF RIFT VALLEY FEVER AND DENGUE FEVER

How frequent do you get bitten by mosquitoes?

- Often
- Sometimes
- Seldom
- Never

Which season/time of the year are mosquitoes common?

What time of the day do mostly you get bitten?

- Morning
- Afternoon
- Evening
- At night(in bed)

Where do you mostly get bitten?

- Indoors
- Bushes
- Watering points
- River
- Conservation areas
- Agricultural plot
- Grazing lands
- Lake
- Other

Specify(other)

Do you think the populations/biting of mosquitoes in this area has changed compared to past years?

- Increased
- No change
- Decreased
- I don't know

What could be the reason for this change?

Present this question if previous answer was increased/decreased otherwise enter NA

What is the mosquito transmitting Rift Valley fever and Dengue fever called?
Allow also local names for mosquito

What are the breeding places of mosquitoes in this area?
Allow respondent to list while you tick. If not on the list use "other" option

- [ ] Discarded tyres
- [ ] Open septic tanks
- [ ] Open water containers (drums, litter)
- [ ] Blocked drainage pipes
- [ ] Rivers
- [ ] Temporary water puddles
- [ ] Bushy vegetation
- [ ] Lakes
- [ ] Swamp
- [ ] Flower pots
- [ ] Other
- [ ] I don't know

Specify(other)

With reference to vegetation, which type of bushes do you associate with mosquitoes?
Allow local names of plants if you cannot translate it immediately

---

Prevention of both Rift Valley fever and Dengue fever

L. GENERAL PREVENTATIVE PRACTISES AGAINST RIFT VALLEY FEVER AND DENGUE FEVER

What do you do to prevent your livestock and yourself from being infected with the two diseases we are discussing here ?

Allow respondent to list while you tick. If not on the list use "other" option

- [ ] Reduce contact with sick looking livestock
- [ ] Wearing protective equipment when dealing with sick animals/persons
- [ ] Separate sick livestock from healthy ones
- [ ] Avoiding grazing/mixing animals in wild animal areas
- [ ] Eliminate wild animals identified as a threat for disease transmission
- [ ] Vaccination
- [ ] Disposing dead animals/arbotus
- [ ] Properly cooking meat and boiling milk
- [ ] Bed net use
- [ ] Use of window screens to prevent mosquito bites
- [ ] Avoid going to places where mosquitoes are abundant
- [ ] Avoid bringing livestock in places where mosquito are abundant
- [ ] Use of mosquito repellant (traditional/modern)
- [ ] Use of mosquito coils
- [ ] Eliminating mosquito breeding places
- [ ] Insecticide spraying indoors and outdoors
- [ ] Using mosquito traps
- [ ] Other
- [ ] I don't know

Specify(other)

Current prevention of Rift Valley fever

M. CURRENT PREVENTATIVE EFFORTS AGAINST RIFT VALLEY FEVER

At a larger scale than your individual action, what is being done in the area to prevent the transmission of RVF to people/animals?

Allow respondent to list while you tick. If not on the list use "other" option

- [ ] Insecticide Spraying (large-scale)
- [ ] Elimination of wildlife identified as a threat
- [ ] Installation of vector traps
- [ ] Fences to reduce contacts between wild/domestic animals
- [ ] Bush/vegetation clearing
- [ ] Vaccination campaigns for animals
- [ ] Vaccination campaigns for people
- [ ] Prophylactic treatments for animals
- [ ] Prophylactic treatments for people
- [ ] Nothing
- [ ] Other

Specify(other)

Who is conducting the measure selected?

The options include but are not limited to NGOs, national government, county government, local authorities

---

Do you think the measures to control the transmission of RVF are helpful in this area?

Measure as selected in previous question of this section

- ○ Strongly agree
- ○ Agree
- ○ Neither agree/disagree
- ○ Disagree
- ○ Strongly disagree

Explain your choice

They should explain their choice in the likert ranking in previous question

---

Current prevention of Dengue fever

N. CURRENT PREVENTATIVE PRACTISES AGAINST DENGUE FEVER

At a larger scale than your individual action, what is being done in the area to prevent the transmission of dengue to people

Allow respondent to list while you tick. If not on the list use "other" option

- [ ] Insecticide Spraying (large-scale)
- [ ] Elimination of wildlife identified as a threat
- [ ] Installation of vector traps
- [ ] Fences to reduce contacts between wild/domestic animals
- [ ] Bush/vegetation clearing
- [ ] Vaccination campaigns for animals
- [ ] Vaccination campaigns for people
- [ ] Prophylactic treatments for animals
- [ ] Prophylactic treatments for people
- [ ] Nothing
- [ ] Other

Specify(other)

Who is conducting the measure selected?

Options include but are not limited to NGOs, national government, county government, local authorities

---

Do you think the measures to control the transmission of dengue are helpful in this area?

Measure as selected in previous question of this section

- ○ Strongly agree
- ○ Agree
- ○ Neither agree/disagree
- ○ Disagree
- ○ Strongly disagree

Explain your choice

They should explain their choice in the likert ranking in previous question

---
