# Supplementary material for: Understanding knowledge, attitude and perception of Rift Valley fever in Baringo South, Kenya: A cross-sectional study
Source: PLOS Glob Public Health. 2023 Sep 12;3(9):e0002195. doi: 10.1371/journal.pgph.0002195 (PMC10497146; doi:10.1371/journal.pgph.0002195)
Supplement: S2 Text — (DOCX) [file pgph.0002195.s003.docx]

**S2 Text**: Socio-economic scores and contributions of household characteristics and assets to the multicorrespondence analysis (MCA)

The scores and the relative contribution assigned to each variable in the multicorrespondence analysis (MCA) is shown in the table. As expected owning a material asset (e.g. television, radio) was associated with a positive score while not owning it resulted in a negative score. This was also the same for house condition where modern and durable construction materials (e.g. brick, iron sheets) were associated with a positive score while traditional ones (e.g. grass, mud) were associated with a negative score. Having access to clean water and basic ablution facilities was associated with a positive score while not having access to these amenities resulted in a negative score. This was also the case for the source of energy used by the household for cooking and lighting where for example electricity were associated with a positive score while firewood was associated with a negative score. Overall, variables with negative score had a negative effect on the socio-economic score and the opposite was true for variables with a positive score.

**Table**: Variable scores and relative contributions to the final MCA

| Variable | Score | Contribution |
| --- | --- | --- |
| Iron sheets roof | 0.164 | 0.8 |
| Thatched roof | -1.726 | 8.433 |
| Cement floor | 1.118 | 11.434 |
| Earth floor | -0.435 | 4.446 |
| Brick & cement wall | 1.150 | 1.873 |
| Brick & mud wall | -0.884 | 0.425 |
| Grass & pole wall | -2.128 | 0.493 |
| Iron sheet wall | 0.226 | 1.296 |
| Mud & pole wall | -1.302 | 8.674 |
| No TV | -0.380 | 3.596 |
| TV | 1.225 | 11.598 |
| No radio | -0.332 | 1.821 |
| Radio | 0.341 | 1.871 |
| Computer | 2.719 | 0.805 |
| No computer | -.009 | 0.003 |
| No smartphone | -0.253 | 1.685 |
| Smartphone | 1.055 | 7.032 |
| Charcoal | 0.780 | 0.596 |
| Firewood | -0.024 | 0.018 |
| Candle | -.0849 | 0.078 |
| Electricity | 1.387 | 3.140 |
| Solar | 0.276 | 1.726 |
| Torch | -1.018 | 8.575 |
| Borehole | 0.289 | 0.907 |
| Harvested rainwater | 0.917 | 0.366 |
| Open source | -0.142 | 0.396 |
| Piped | 0.882 | 0.678 |
| Spring | -1.644 | 1.765 |
| Protected well | -2.071 | 0.934 |
| Pit with slab | 0.832 | 3.765 |
| No toilet | -0.5 | 5.22 |
| Pit toilet | 0.937 | 5.548 |


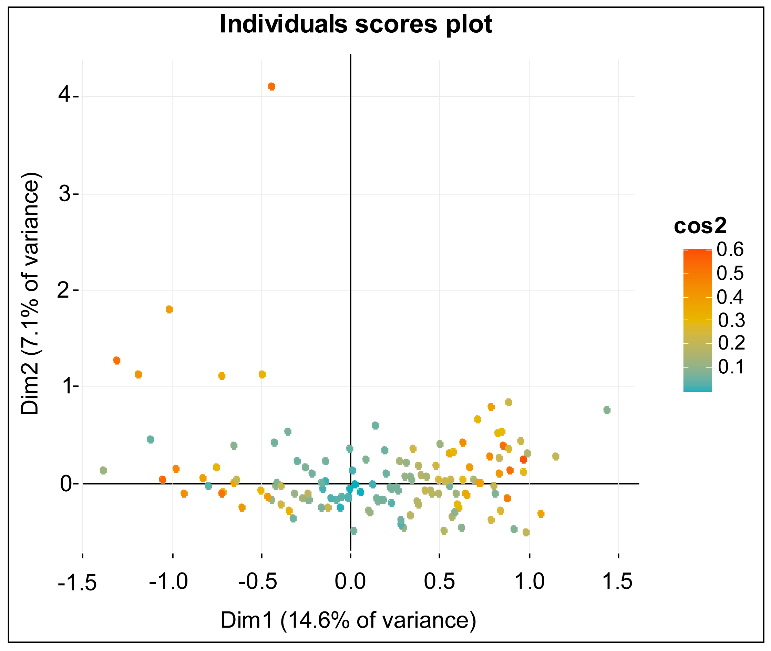


**Figure**: Individual factor map showing the results of MCA on household condition and assets on two dimensions
