# Supplementary figures and images for: Understanding knowledge, attitude and perception of Rift Valley fever in Baringo South, Kenya: A cross-sectional study
Source: PLOS Glob Public Health. 2023 Sep 12;3(9):e0002195. doi: 10.1371/journal.pgph.0002195 (PMC10497146; doi:10.1371/journal.pgph.0002195)

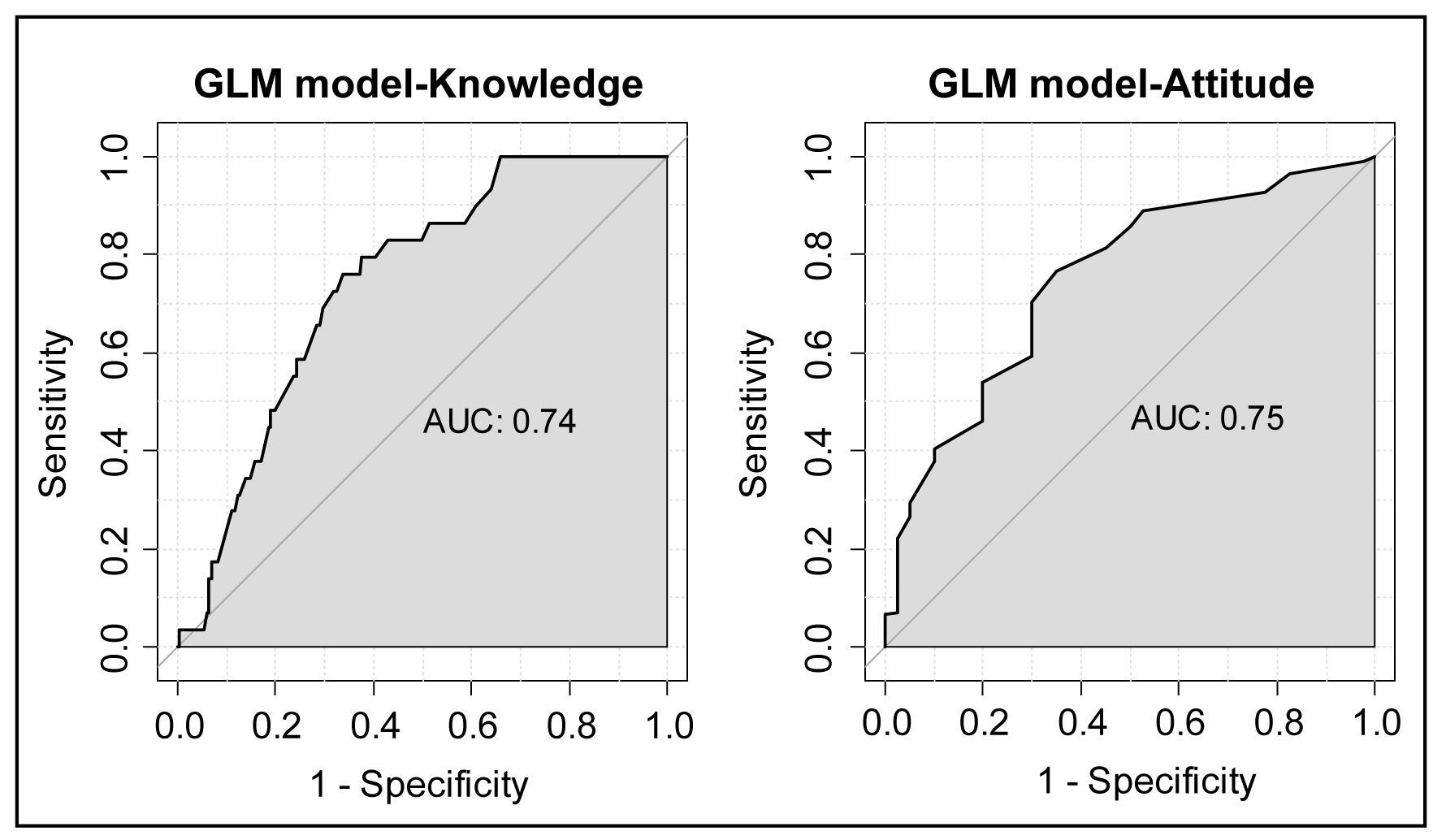

Supplement: S1 Fig — (TIF) [file pgph.0002195.s004.tif]
