# Supplementary material for: Understanding knowledge, attitude and perception of Rift Valley fever in Baringo South, Kenya: A cross-sectional study
Source: PLOS Glob Public Health. 2023 Sep 12;3(9):e0002195. doi: 10.1371/journal.pgph.0002195 (PMC10497146; doi:10.1371/journal.pgph.0002195)
Supplement: Attachment — Submitted filename: Review_PGPH-D-23-00614.pdf [file pgph.0002195.s005.pdf]

Dear authors,

The authors investigated risk factors of RVF using a structured questionnaire. Major and minor concerns are listed below.

1. In line 121, it was stated that the "participants were selected randomly". Please specify which random sampling were applied during the study.

2. In line 121-122, "The total number of respondents was proportionally constituted based on the population and number of households in each location." It is unclear here. Please briefly describe how it proportionally constituted to the population as well as number of households in each location.

3. line 124-125, "At each household the head or another person from that household above the age of 18 were interviewed and data were recorded". How many people were interviewed from each household? If more than one person, the why only head of the household were interviewed not all the participants? It is a knowledge, attitude and perception study, therefore, if only the head of the household was interviewed then there would be bias in this study.

4. No information regarding the correlation analysis between the continuous independent variables was mentioned in the data analysis section. Did the author try to find out the correlation matrix among continuous independent variables? If yes, then what was cut off value to identify high correlated variables, and what was the basis of selection of variables from highly correlated variables?

5. The author did not analyze the univariable association between independent and dependent variables. Is there any specific reason for not doing univariable analysis?

6. After fitting the final model in multivariable analysis, did the author try to find out if there was any confounder or not? How was the confounder checked in the final model?

7. There was no information regarding the interaction of the variables from the final model. Did the author check any interactions of significant variables from the final model?

8. How the goodness of the fit of final model was checked? Please specify which test was used to find out the overall goodness of fit of the final model.

9. How the predictive ability of the model was identified? Generally, the Receiver Operating Characteristics (ROC) curve helps to identify the power of the final model. Please add this to the manuscript.
