# Supplementary material for: Understanding knowledge, attitude and perception of Rift Valley fever in Baringo South, Kenya: A cross-sectional study
Source: PLOS Glob Public Health. 2023 Sep 12;3(9):e0002195. doi: 10.1371/journal.pgph.0002195 (PMC10497146; doi:10.1371/journal.pgph.0002195)
Supplement: Attachment — Submitted filename: Response to Reviewers.docx [file pgph.0002195.s006.docx]

**Response to reviewers** *(Note that the line numbers in this document refer to the Revised Manuscript with Track Changes)*

**We want to thank all the reviewers for their comments which have significantly improved the manuscript. We have addressed these comments in the *Revised Manuscript with Track Changes* and in the responses below.**

***Editor and journal requirements***

1. Please include a complete copy of PLOS’ questionnaire on inclusivity in global research in your revised manuscript.

**Done**

2. Please amend your detailed Financial Disclosure statement. This is published with the article. It must therefore be completed in full sentences and contain the exact wording you wish to be published.

a. State the initials, alongside each funding source, of each author to receive each grant.

b. If any authors received a salary from any of your funders, please state which authors and which funders.

**Done**

3. In the online submission form, you indicated that "The dataset generated and analysed in this study can be made available from the corresponding authors on reasonable request". All PLOS journals now require all data underlying the findings described in their manuscript to be freely available to other researchers, either 1. In a public repository, 2. Within the manuscript itself, or 3. Uploaded as supplementary information.

**The data is available from the webserver of the Center for Development Research (ZEF).** https://www.zef.de/fileadmin/downloads/Marigat_RVF_KAP_study.xlsx.

**Reviewer #1**

1. In line 121, it was stated that the “participants were selected randomly”. Please specify which

random sampling was applied during the study.

**The random sampling of respondents was performed in each of the selected villages hence it is stratified random sampling. The sentence has been edited in line 127-128 to reflect this as follows: “*Complete households’ lists were obtained from village heads through the location chiefs and used to select respondents using stratified random sampling.”***

2. In line 121-122, “The total number of respondents was proportionally constituted based on the

population and number of households in each location.” It is unclear here. Please briefly describe

how it proportionally constituted to the population as well as number of households in each

location.

**The proportions were actually based on the number of households which was our sampling unit. A brief description has been added in line 128-135:** **“*The total number of respondents was proportionally constituted based on the number of households in each location.*** ***According to available recent census data there are 296 households in Kapkuikui, 672 in Loboi, 954 in Ng’ambo, 967 in Salabani and 707 in Sandai giving a total of 3596 households in the five locations [28]. Therefore, respondents from Kapkuikui constituted 8.2% of the study population while those from Loboi, Ng’ambo, Salabani and Sandai made up 18.7%, 26.5%, 26.9%, and 19.7% of the study population respectively.”***

3. line 124-125, “At each household the head or another person from that household above the

age of 18 were interviewed and data were recorded”. How many people were interviewed from

each household? If more than one person, the why only head of the household were interviewed

not all the participants? It is a knowledge, attitude and perception study, therefore, if only the

head of the household was interviewed then there would be bias in this study.

**Indeed, only one person was interviewed from a selected household (the head and if not present another member above the age of 18). In most communities the household is a single unit and the head is in charge and is expected to be more knowledgeable of all aspects affecting the household. More so, in the context of Rift Valley fever which is a disease that has a long inter-outbreak period and has significant socio-economic impact. Administering the questionnaire to 2 or 3 members at each household is time consuming and would necessitate keeping the whole household at home from their daily routines for extended periods. We believe the method we used has minimal bias as highlighted by its use in several past KAP studies.**

4. No information regarding the correlation analysis between the continuous independent

variables was mentioned in the data analysis section. Did the author try to find out the correlation

matrix among continuous independent variables? If yes, then what was cut off value to identify

high correlated variables, and what was the basis of selection of variables from highly correlated

variables?

**We have added the information in the methods section in line 200-202:** ***“Multicollinearity among all the variables was checked using the generalised variance inflation factors (GVIFs) for both continuous and categorical variables where a value below 10 is considered acceptable.”***

**The results of this analysis are shown as a footnote below each of the final models presented in Table 4 *(“The independent variables had GVIFs less than 1.2 indicating absence of serious multicollinearity.”*) and Table 5 *(“The independent variables had GVIFs less than 1.1 indicating absence of serious multicollinearity.”***)

5. The author did not analyze the univariable association between independent and dependent

variables. Is there any specific reason for not doing univariable analysis?

**This was omitted in error but the univariate analysis has been added to each of the revised Tables 4 and 5.**

6. After fitting the final model in multivariable analysis, did the author try to find out if there was

any confounder or not? How was the confounder checked in the final model?

**No confounding was detected. Confounders were checked by testing the effect of biologically plausible interactions on the two models for knowledge and attitude. The models with the interactions however were not any better than the simpler models based on the likelihood ratio test, hence they were discarded.**

7. There was no information regarding the interaction of the variables from the final model. Did

the author check any interactions of significant variables from the final model?

**Yes, we checked for interactions and these did not seem to improve our models. See above comment.**

8. How the goodness of the fit of final model was checked? Please specify which test was used to

find out the overall goodness of fit of the final model.

**The Hosmer-Lemeshow test was used to check the goodness of fit of the two models. We have added this information in the Methods section in line 202-205: *“The Hosmer-Lemeshow goodness of fit test was carried out on the final models to assess if they fitted the observed data adequately. In this test the null hypothesis is that there is no difference between the observed data and that predicted by the model.”***

**The results of this analysis are shown in the footnotes of Tables 4 and 5. These indicate that the models are a good fit for the data. Table 4: *“The Hosmer-Lemeshow goodness of fit test gave a chi-square value of 1.4063, df = 8 and a p-value of 0.994 suggesting that the model fits the data well.”* Table 5*: “The Hosmer-Lemeshow goodness of fit test gave a chi-square value of 7.8112, df = 8 and a p-value of 0.4521 suggesting that the model fits the data well.”***

9. How the predictive ability of the model was identified? Generally, the Receiver Operating

Characteristics (ROC) curve helps to identify the power of the final model. Please add this to the

manuscript.

**This information has been added to the Methods section in line 205-207. *“Receiver operating curves (ROC) with area under the curve (AUC) values were used to show the models’ predictive ability. Area under the curve values between 0.7 and 0.8 are considered acceptable while 0.8 to 0.9 is excellent, and more than 0.9 outstanding [33].”* The AUC values have been added to the footnotes of Table 4 *(“The area under the curve for the model was 0.74 which is an acceptable predictive power.”*) and Table 5 *(“The area under the curve for the model was 0.75 which is an acceptable predictive power.”*) that present the two models. The curves are also shown in Supporting file 4.**

**Reviewer #2**

1. Firstly, I would recommend structuring the abstract according to the traditional format of background, methods, results, and conclusion. This standard organization can greatly improve the overall readability of the abstract, allowing readers to quickly grasp the key aspects of your research.

**We have put in some paragraphs in the abstract to highlight the structure of the abstract as journal requirements do not permit explicit labelling of the sub-heading’s ‘background’, ‘methods’ etc.**

2. Secondly, I suggest reorganizing the presentation of your results to enhance clarity and flow. By ensuring a logical progression and grouping related findings together, readers will find it easier to follow your research and understand the implications of your results.

**As a consequence of the extended revision of the results session, a reflection of the three helpful reviews, we believe that with this the clarity and flow of the section has considerably been improved.**

3. Additionally, I encourage you to consider rephrasing sentences throughout the manuscript to achieve greater clarity and conciseness. For example, in sentence 41, you can rephrase it as "Rift Valley fever (RVF) is a zoonotic disease caused by the Rift Valley fever virus (RVFV)." Shortening sentences will make the content more accessible and facilitate a better understanding of the subject matter.

**We agree with this suggestion and have shortened the more elongated sentences**

**Reviewer #3**

Abstract “86% had a positive attitude”

- I know this is just an abstract so hard to elaborate, but would help to add little more info so the reader knows what “positive attitude” means. They like RVF? They are confident they understand it?

**A brief explanation of what attitude entails has been added in the abstract in line 28-30:** **“*Nevertheless, 86% recognized the threat it posed and knew the appropriate action to take in suspected human and livestock cases (positive attitude).”***

Line 46: In the first sentence of the abstract you say RVF causes high morbidity and mortality in humans but in intro line 46 you say it progresses to sever symptoms “in rare cases.” This feels contradictory to describe it in both ways.

**The first sentence of the abstract has been modified to avoid the contradiction with the introduction:** ***“Rift Valley fever (RVF) is a mosquito-borne viral hemorrhagic disease that affects humans and livestock.”***

Line 55: “signifying increased receptivity.” The word receptivity is confusing wording, as it suggests acceptance of the virus. I would recommend replacing with something like “signifying increased potential for transmission in most parts of the country.”

**The statement has been rephrased as suggested in line 56-58: *“The number of counties affected have drastically increased, signifying increased potential for transmission in most parts of the country”***

Line 62: Most people don’t know what solanchak soils are. Maybe include a brief description of why that type of soil is conducive to RVF outbreaks?

**A brief description of this type of soil has been added in line 66-68:** ***“There are also clay-rich, impermeable soils with high surface water retention properties (solanchak soils). Considering the presence of these factors, the virus is likely to pose a constant threat in the County.”***

Line 119: “In each of the locations, eight villages were randomly selected with four villages each per sub-location.” I do not understand this sentence. Were 8 villages randomly selected, and then 4 of those 8 were decided on? What are the sub-locations? It would be helpful if this were more clear.

**To make it clear this sentence has been edited to appear as follows in line 123-125:** ***“In each of the locations, eight villages were randomly selected. Each location is made up of 2 sub-locations therefore each sub-location contributed 4 villages.”***

Line 141: I personally recommend against defining acronyms/abbreviations that are not extremely well established and understood by the general field. While writing TLU helps with word count, most readers will forget what that means and you will lose impact when reporting the results. For example, while I was reading I got lost trying to go back up to find the definition, and then go back to where I was reading. If make the change then you’ll need to go through and change TLU to tropical livestock units throughout.

**As suggested TLU and SEI has been written out in full throughout the manuscript.**

Line 142: As above, I recommend just writing out socio-economic index instead of SEI; it makes life a lot easier for the reader.

**Done**

Line 145: I am unfamiliar with this specific SEI index and the method (MCA) used to calculate the index. I think it would be really helpful if the authors added a brief explanation of how this works. Are there weights assigned to each of the variables, and is the weight determined by the authors or is weight determined by MCA through some sort of regression?

**A brief explanation of the method has been added in line 162-168. This is in addition to the information in Supporting file 2:** ***“Multiple correspondence analysis (MCA) is a reduction method that is an extension of Principal component analysis (PCA) (for continuous data). It is used to analyze relationships in multi-dimensional categorical population data by converting it into fewer dimensions that explain much of the variation within a study population. It reveals the quantity and quality of the contribution of each variable to these dimensions on a numerical scale. The weighted contribution is then used to generate a numerical variable for each individual.”***

Line 159: This is the first mention of attitude and it is unclear what attitude means and why attitude is being explored. It is very clear to me why knowledge is important, but not clear my attitude is important. It would be nice if there were a sentence or two explaining the importance of attitude in preventing RVF outbreaks.

**We have added a brief explanation on why attitude is important and how it was measured in this study in line 181-187:** **“*****Attitude was determined from how the respondents perceive the threat (serious, mild, not important etc.) posed by RVF and the action they would take in suspected human and livestock cases (seek professional medical/veterinary advice, use traditional herbs, do nothing etc.). Attitude is an important factor in the success of community-based awareness programmes and decision-making. Communities with a positive attitude are more likely to follow and institute prevention measures during RVF outbreaks compared to those with a negative attitude****.***”**

Line 179-181: “Most of the household heads were aged between 30-39 years 71/300

180 (23.7%), had an education up to primary level 149/300 (49.7%), were married 231/300 (77%)

181 and protestants 181/300 (60.3%).” The grammar in this sentence makes it hard to understand what numbers are associated with what variables. Can you please revise?

**Sentence revised in line 219-222:** **“Most (23.7%; 71/300) of the household heads were aged between 30-39 years while 49.7% (149/300) had an education up to primary level. The majority (77%; 231/300) were married and 60.3% (181/300) belonged to a protestant religion.”**

Line 189-191: are relatives considered extended family or does that include friends/neighbors too? If it includes neighbors/friends, maybe just say “12% reported knowing someone who had RVF.” Also, there is a difference between having someone in the household with previous exposure and just knowing someone. Maybe report them separately; if someone in their house had been sick they probably are going to be much more aware compared to just knowing someone from a neighboring village.

**The question specifically referred to a household member and the statement has been edited in line 231-234 to reflect this:** ***“In the five locations, 12% (36/300) reported having a member of the household that had suffered from RVF previously, while 23% (69/300) reported having livestock that had suffered from RVF before.”***

Line 194: When you report Tropical Livestock Units, I recommend saying whether they had 4 or more, or less than 4, given the overall average was 3.6.

**Done**

Line 227: Why are the numbers not reported for each location? If you can’t share the data in the manuscript for fear of demeaning these communities, it would be helpful to know if knowledge regarding each topic was consistent across the locations. For example, you could add the range of values across locations to show that some places differ in levels of understanding. Would be very helpful in real life for stakeholders to have this info of course.

**Numbers have been added for each location in addition to the total tally in both Tables 2 and 3.**

Line 235-237: The invasive species question is very interesting. Can you add data on how many implicated this type of vegetation? (not required)

**Data added**

Line 292: Before going into results from the adjusted multivariate regression model, need to explain which covariates were selected for inclusion. At least, covariate selection was what I was expecting based on the statement in lines 169-171.

**The selection of covariates has been added to the Methods section in line 191-197: “*The matrix of successes and failures was used as the dependent variable initially for univariate analysis and then multivariate analysis for overall knowledge of RVF and attitude. A logistic regression model was fitted to the data to determine the association between the independent and dependent variables. All the factors with p-value less than 0.2 in the univariate analysis were included in the multivariate model for both overall knowledge and attitude.”* The results of univariate analysis have been added to the revised Tables 4 and 5.**

Line 305: Again, it is confusing what a positive attitude means. This should be explained in the methods.

**Done. See the Method section in line 181-187**

Line 313-316: add (N=xx) for each of these. Do a considerable proportion of people believe they should mix sheep blood with faeces? This would be concerning. OR is this just a single person?

**There were 1 or 2 respondents who gave these answers. The data has been added in line 371-374: *“Other actions mentioned when suspecting infection included “do nothing” (“It’s an animal disease”) (0.6%; 2/300), buy medication from the pharmacy (“while waiting to get to hospital”), (0.6%; 2/300) visiting traditional healers (0.3;1/300 and drinking sheep blood mixed with its faeces (0.6%; 2/300).”***

Line 323-324: what proportion hinted at eating the animals?

**Only 4 respondents mentioned this. The data has been added to the sentence in line 381-383: *“Burying carcasses was the most mentioned 208/300 (69.3%) mode of disposing animals that die of suspected RVF while other respondents hinted at eating the meat or giving it to their dogs (1.3%; 4/300).”***

Line 331-336: this will be much more clear and interpretable when you explain what positive attitude means.

**Explained in the Methods section**

Line 442: can you please add a citation for timing and location that bites occur

**Added in text (line 505) and the bibliography [45]:** “**Rund SSC, Labb LF, Bene OM, Duf GE. Artificial light at night increases *Aedes aegypti* mosquito biting behaviour with implications for arboviral disease transmission. Am J Trop Med Hyg 2020; 103:2450–2. https://doi.org/10.4269/ajtmh.20-0885.**”

Line 349: I don’t see any discussion about the multivariate model results. This leads to an important question: what do we learn from running a multivariate model that we don’t learn from univariate analysis? In the methods it would be nice to have an explanation of why the decision was made to do an adjusted model, and then add discussion as necessary based on your justification. (Important)

**We are not clear on this comment because it is the multivariate model results that are discussed in detail. However, we are assuming the reviewer means they *don’t see a discussion on the univariate analysis*. As per standard practice, the univariate analysis is mainly exploratory to identify variables that are relevant for further analysis. While the results of univariate analysis are mentioned, the adjusted model is preferred when making inferences because it analyses the relationship between a variable and the outcome in the presence of other selected predictors thereby enhancing its predictive value.**
