# Supplementary material for: Understanding knowledge, attitude and perception of Rift Valley fever in Baringo South, Kenya: A cross-sectional study
Source: PLOS Glob Public Health. 2023 Sep 12;3(9):e0002195. doi: 10.1371/journal.pgph.0002195 (PMC10497146; doi:10.1371/journal.pgph.0002195)
Supplement: Attachment — Submitted filename: Additional editor comments.docx [file pgph.0002195.s007.docx]

- Abstract: You have mentioned “only 9.6% attained at least half of the overall knowledge score on RVF”. The term “overall knowledge score” is poorly measurable and understandable. I think that it “total score”. Please clarify this.
- Abstract: You suggested to perform regular awareness campaigns during inter-outbreak period. It is better to perform awareness campaigns regular basis all the time. During outbreak period it is also important.
- In the multivariate logistic regression, the association between overall knowledge of RVF and factors was identified. It is important to define the “overall knowledge” (e.g. participants who scored >5 out of 10).
- Table 4: why the variable “Tropical livestock units” was not included in multivariate logistic regression
- Table 5: why the variable “Gender” was not included in multivariate logistic regression
- Line number 419: references [6,34] stands for what information?
- I believe that every study has some limitations. It is important to mention limitation at the end of discussion section if the study had few limitations.
