# Supplementary material for: Understanding knowledge, attitude and perception of Rift Valley fever in Baringo South, Kenya: A cross-sectional study
Source: PLOS Glob Public Health. 2023 Sep 12;3(9):e0002195. doi: 10.1371/journal.pgph.0002195 (PMC10497146; doi:10.1371/journal.pgph.0002195)
Supplement: Attachment — Submitted filename: Response to reviewers.docx [file pgph.0002195.s008.docx]

**Response to reviewers** *(indicated line numbers refer to the Revised Manuscript with Tracked Changes)*

**We are grateful for the invaluable input of the editor and the reviewers which has greatly improved the outlook and content of this manuscript. We have addressed the latest comments raised by the editor below.**

1. Please review your reference list to ensure that it is complete and correct. If you have cited papers that have been retracted, please include the rationale for doing so in the manuscript text, or remove these references and replace them with relevant current references. Any changes to the reference list should be mentioned in the rebuttal letter that accompanies your revised manuscript. If you need to cite a retracted article, indicate the article’s retracted status in the References list and also include a citation and full reference for the retraction notice.

**The reference list has been checked and minor edits done to accurately fit journal referencing style. While none of the articles we cited have been retracted we replaced reference [1] in text (line 43) and in the reference list. This is to highlight the fact that currently Rift Valley fever virus is classified in the family Phenuiviridae. The new citation is as follows:**

**Wright D, Kortekaas J, Bowden TA, Warimwe GM. Rift Valley fever: biology and epidemiology. J Gen Virol. 2019:1187–99. https://doi.org/10.1099/jgv.0.001296.**

- Abstract: You have mentioned “only 9.6% attained at least half of the overall knowledge score on RVF”. The term “overall knowledge score” is poorly measurable and understandable. I think that it “total score”. Please clarify this.

**The statement has been corrected in the abstract in line 27-28. Throughout the manuscript ‘*overall*’ has been replaced with ‘*total’* where appropriate. “*Out of the 300 respondents, 80% had heard about the disease, however, only 9.6% attained at least half of the total knowledge score on RVF*”.**

- Abstract: You suggested to perform regular awareness campaigns during inter-outbreak period. It is better to perform awareness campaigns regular basis all the time. During outbreak period it is also important.

**Sentence edited to reflect that in the abstract in line 35-36. “*This calls for regular awareness campaigns*”.**

- In the multivariate logistic regression, the association between overall knowledge of RVF and factors was identified. It is important to define the “overall knowledge” (e.g. participants who scored >5 out of 10).

**A brief explanation has been added in the Methods in line 189-190. “*Participants who scored at least 50% of the total scores were deemed to have better knowledge and attitude to RVF respectively*”.**

- Table 4: why the variable “Tropical livestock units” was not included in multivariate logistic regression

**It was included in the analysis but it was dropped from the final model. Using the *stepAIC* method of final model determination the model without the variable ‘*Tropical livestock units*’ was better than the one when it is present.**

- Table 5: why the variable “Gender” was not included in multivariate logistic regression

**The same explanation given above for Table 4 also applies here for ‘*Gender*’.**

- Line number 419: references [6,34] stands for what information?

**It seems the citation was misplaced and has been removed in line 427.**

- I believe that every study has some limitations. It is important to mention limitation at the end of discussion section if the study had few limitations.

**Some limitations to the study are mentioned at the end of the discussion in line 547-557.**

**“*The findings of this study should be construed in light of the following limitations: The last major RVF outbreak that affected Baringo County occurred about 13 years ago and hence the study could have been affected by recall bias in the participants. While it is important for the participants to have good knowledge on RVF it is equally important to translate the knowledge into good practices. Some bias could be introduced when this ‘good practice’ is self-reported as in this study rather than observed. It is a challenge to accurately determine if there is history of RVF occurrence in the household (human or livestock cases) as some cases may have been suspected but not diagnosed by a clinician or veterinarian. Nonetheless this study provides a useful perspective on the current level of knowledge on RVF and its determinants in Baringo County paving way for remedial action*”.**
